# Psychiatric Adverse Effects From Prucalopride in a Medically Complex Adolescent

**DOI:** 10.1155/crpe/6639748

**Published:** 2025-04-01

**Authors:** Clare Hawkes, Jonathan J. Chandran, Kasia Kozlowska

**Affiliations:** ^1^School of Psychological Sciences, University of Tasmania, Launceston, Australia; ^2^The Department of Adolescent and Young Adult Medicine, Westmead Hospital, Sydney, Australia; ^3^The Department of Psychological Medicine, The Children's Hospital at Westmead, Sydney, Australia; ^4^Brain Dynamics Centre, Westmead Institute for Medical Research, Westmead, New South Wales, Australia; ^5^Discipline of Psychiatry and Discipline of Child & Adolescent Health, University of Sydney Medical School, Sydney, Australia

**Keywords:** adolescent, adverse effects, paediatrics, prucalopride, psychiatry

## Abstract

**Background:** Prucalopride is a highly selective, 5-HT4 receptor agonist that can be used for the treatment of chronic constipation in individuals for whom laxatives fail to provide adequate relief. The current case study describes adverse neuropsychiatric symptoms following the administration of prucalopride in a 15-year-old female with a complex physical and mental health history to manage chronic constipation.

**Case Summary:** A single 2 mg dose of prucalopride was prescribed to a 15-year-old female to manage her chronic constipation due to a diagnosis of autoimmune enteric neuropathy. Following the oral administration of prucalopride, the patient started experiencing visual and auditory hallucinations, along with suicidal ideation. Prucalopride was ceased, with the patient receiving psychopharmacology and psychological intervention to address the acute onset psychiatric symptoms.

**Practical Implications:** To our knowledge, this is the second documented case of acute onset neuropsychiatric symptoms following the administration of prucalopride. Clinicians should be aware of this possible side effect, particularly if considering administering prucalopride in patients with neurodevelopmental and psychiatric comorbid histories. Increased supervision and monitoring is recommended in these patients if prucalopride is administrated.

## 1. Introduction

Prucalopride is a highly selective, 5-HT4 receptor agonist that has shown promise in treating a range of gastrointestinal (GI) disorders [1] including the treatment of chronic constipation in individuals in whom laxatives have failed to provide adequate relief [2]. Selective agonism at the 5-HT4 receptors facilitates the release of neurotransmitters in the GI prokinetic reflex pathways, including 5-HT4. The increase of 5-HT4 results in a dual process. Enteric cholinergic neurons are stimulated. These neurons produce acetylcholine that assists in smooth muscle contraction. Inhibitory enteric (nitrergic) neurons are also stimulated. These neurons release of nitric oxide and assist in smooth muscle relaxation [1]. Prucalopride was approved for use at a dose of 2 mg/day by the European Medicine Agency in 2009, the Food and Drug Administration (FDA) in 2018 and Australian Therapeutic Goods Administration (TGA) in 2022. Within an adult population, prucalopride has been documented as having positive effects on gut peristalsis [3]. There are ongoing research studies looking at 5-HT4 receptor agonists as potential treatments for mood and cognitive problems [3]. Less is known about the efficacy and possible side effects of prucalopride in a paediatric population [1]. While research suggests that prucalopride has better safety and side effect profile when compared to other medications used in the treatment for constipation—such as cisapride and tegaserod [4]—emerging evidence from retrospective data suggests that potential mood alterations may occur following the administration of prucalopride in paediatric patients [1, 5]. In a retrospective study (*n* = 71), Hirsch et al. [5] reported that potential side effects include exacerbation of pre-existing mood disorders (including anxiety and depression) (*n* = 4), new onset suicidal ideation (*n* = 2) and onset of psychosis (*n* = 1) [5]. Despite these potentially concerning adverse side effects being documented, the retrospective nature of the data limits the level of clinical details associated with the cases. In the adult literature, a previous case study has reported the development of neuropsychiatric symptoms following prucalopride administration in a 61-year-old woman for the management of chronic constipation [6]. We present a second case study with adverse psychiatric symptoms following administration of prucalopride, this time in an adolescent female.

## 2. Case Presentation

A 15-year-old adolescent female was prescribed a single 2 mg dose of prucalopride to manage her chronic constipation.

The adolescent had a history of complex gut issues and was diagnosed with an autoimmune enteric neuropathy at 14 years old. Initial management consisted of two treatments with intravenous immunoglobulin (IVIG) and oral steroids. After this treatment was unsuccessful, she had more localised treatments for her gut including aperients (including osmotic and stimulant laxatives and macrolide antibiotics targeting motilin receptor agonists), the use of suppositories, enemas and bowel preparation and regular bowel washouts.

Following the oral administration of the prucalopride (a single dose of 2 mg at 7 p.m. in the evening), the adolescent began to experience distressing psychiatric symptoms: visual and auditory hallucinations (seeing a persistent dark male figure in her hospital room telling her to hurt herself), along with suicidal ideations. She sat on her hospital bed slumped forward responding to, and frightened by, the hallucinations. On assessment by the attending psychiatrist, she reported feeling worried that she might be ‘going mad'. The prucalopride was ceased. The patient was started on 25 mg of quetiapine three times a day to reduce the psychiatric symptoms and to assist in managing her distress [7]. A traffic-light safety plan was implemented to monitor the suicidal ideation and need for one-to-one supervision during the day [8]. The symptoms disappeared 4 days later. The quetiapine was ceased 6 days later.

The adolescent also had a complex neurodevelopmental and psychiatric history. She was diagnosed with autism spectrum disorder (ASD, Level 2) and attention deficit hyperactivity disorder (ADHD). As part of her ASD, she had irritability and frequent episodes of emotional dysregulation and was medicated with the atypical antipsychotic paliperidone (6 mg daily) [9, 10]. In addition, the patient also had a history of anorexia nervosa (restrictive subtype but weight restored, onset at 13 years of age) and major depression (onset at 13 years of age) with intermittent suicidal ideation. She was medicated with fluvoxamine 125 mg daily for her depression. She also had multiple emotion-regulation strategies that she implemented when she became stressed or dysregulated.

Importantly, the adolescent had never experienced any prior psychotic phenomenon. Given the profound impact that her chronic constipation had on her lifestyle, and the multiple therapies tried in the past, the adolescent was anxious but hopeful that prucalopride would be the medication that would finally help manage her symptoms. Her anxious mental state was noted several days prior to the administration of the prucalopride.

## 3. Discussion

The previous adverse event case report by Carnovale et al. [6] discussed the complex interactions between the multiple neural pathways (serotonergic, dopaminergic and GABAergic) that may potentially cause neuropsychiatric symptoms with the addition of a 5-HT4 receptor agonist. In this case, the adolescent had a complex psychiatric and neurodevelopmental history that potentially made her vulnerable to the adverse side effects of prucalopride. Psychotic experiences in individuals with ASD are also well documented in the literature, with an increased risk noted in individuals with comorbid ADHD [11, 12]. In this case, the adolescent's ‘sensitive brain' with an atypical response to normal environmental provocations may have been triggered into an overactivated state through multiple stimuli, including through the addition of prucalopride, with its' strong serotonergic response/action. In addition to changes in brain function in the context of neurodevelopmental disorders, there are also neuromodifications caused by mental health disorders, including changes in brain signalling and distribution and concentration of neurotransmitter binding sites, including 5-HT2A and 5-HT4 [13].

The anxious mental state of the adolescent prior to the prucalopride administration may have put her brain into an activated state. Comorbid anxiety has been reported in patients with psychosis and it is often an early symptom in psychosis [14]. Potentially, the addition of the 5-HT4 receptor agonist tipped the adolescent's brain into an even more overactivated state, thereby causing her constellation of symptoms.

Finally, the co-administration of the selective serotonin reuptake inhibitor (SSRI)—fluvoxamine 125 mg daily—and the 5-HT4 receptor agonist may have increased her serotonin load [15]. While this may have not caused the hallucination symptoms, it may have increased her risk of suicidality, as per previous literature reported with SSRI treatment.

Emerging research [1, 5] has identified similar neuropsychiatric symptoms following the administration of prucalopride. This suggests caution when prescribing prucalopride in paediatric patients, particularly if there is a pre-existing history of a psychiatric disorder. That said, comorbid psychiatric conditions have been identified as being relatively high in paediatric patients experiencing constipation [5]. This highlights the importance of close supervision and monitoring for emerging, or escalating, psychiatric symptoms in children and adolescences prescribed prucalopride, to ensure safety and well-being.

## 4. Conclusion

In light of this case—and emerging data from paediatric research [1, 5]—we suggest that clinicians should discuss the rare yet debilitating (and potentially life threatening) risk of neuropsychiatric symptoms associated with this prucalopride, with the patient and their family. We also suggest considering the neurodevelopmental and psychiatric comorbid history of the patient and increasing supervision and monitoring if prucalopride is administered.

## Data Availability

Data sharing is not applicable to this article as no new data were created or analysed in this study.
